# Chemotherapy-Induced Neuropathy and Diabetes: A Scoping Review

**DOI:** 10.3390/curroncol28040273

**Published:** 2021-08-19

**Authors:** Mar Sempere-Bigorra, Iván Julián-Rochina, Omar Cauli

**Affiliations:** 1 Department of Nursing, University of Valencia, 46010 Valencia, Spain; marsembi@outlook.es (M.S.-B.); ivan.julian@uv.es (I.J.-R.); 2 Frailty Research Organized Group (FROG), University of Valencia, 46010 Valencia, Spain

**Keywords:** toxicity, side effects, cold sensitivity, autonomic dysfunction, regimens, cytostatic drugs

## Abstract

Although cancer and diabetes are common diseases, the relationship between diabetes, neuropathy and the risk of developing peripheral sensory neuropathy while or after receiving chemotherapy is uncertain. In this review, we highlight the effects of chemotherapy on the onset or progression of neuropathy in diabetic patients. We searched the literature in Medline and Scopus, covering all entries until 31 January 2021. The inclusion and exclusion criteria were: (1) original article (2) full text published in English or Spanish; (3) neuropathy was specifically assessed (4) the authors separately analyzed the outcomes in diabetic patients. A total of 259 papers were retrieved. Finally, eight articles fulfilled the criteria, and four more articles were retrieved from the references of the selected articles. The analysis of the studies covered the information about neuropathy recorded in 768 cancer patients with diabetes and 5247 control cases (non-diabetic patients). The drugs investigated are chemotherapy drugs with high potential to induce neuropathy, such as platinum derivatives and taxanes, which are currently the mainstay of treatment of various cancers. The predisposing effect of co-morbid diabetes on chemotherapy-induced peripheral neuropathy depends on the type of symptoms and drug used, but manifest at any drug regimen dosage, although greater neuropathic signs are also observed at higher dosages in diabetic patients. The deleterious effects of chemotherapy on diabetic patients seem to last longer, since peripheral neuropathy persisted in a higher proportion of diabetic patients than non-diabetic patients for up to two years after treatment. Future studies investigating the risk of developing peripheral neuropathy in cancer patients with comorbid diabetes need to consider the duration of diabetes, cancer-induced neuropathic effects per se (prior chemotherapy administration), and the effects of previous cancer management strategies such as radiotherapy and surgery.

## 1. Introduction

Diabetes mellitus and cancer are among the four most common chronic diseases, and are two of the leading causes of death worldwide [1]. Treatment of cancer patients with chemotherapy is influenced by multiple aspects, including neuropathy induced by chemotherapy drugs their self [2,3,4]. Chemotherapy-induced neuropathy is a significant source of morbidity in cancer patients, with high incidences (Table 1) and can be a dose-limiting side effect for many classes of chemotherapy drugs [5]. Diabetes mellitus is a commonly encountered comorbidity among patients with solid tumors [6,7,8]. Diabetes mellitus and its accompanying metabolic syndrome have been shown to correlate with the development and outcomes of a number of solid tumors [6,7,9]. Outcomes are often worse in cancer patients who also have diabetes [3]. It is well known that high blood glucose can damage peripheral nerves [10,11]; as such, patients with diabetes who are treated with chemotherapy drugs may be at greater risk of developing chemotherapy-related peripheral neuropathy [12]. Despite the high prevalence of neuropathy among cancer and diabetes patients, little is known about neuropathic symptoms among cancer patients with comorbid diabetes. Because peripheral neuropathy is commonly observed in patients with diabetes, most studies of chemotherapy-induced peripheral neuropathy exclude patients with diabetes [5]. An analysis of the trial E1199, which included 5052 patients with axillary node-positive or high-risk, node-negative breast cancer showed that patients who received adjuvant taxanes containing therapy, glycemic instability and obesity were associated with an increased risk of neuropathy. However, information regarding a pre-existing history of diabetes was not available in this study [13].

Although there is considerable evidence that patients with pre-existing symptoms of diabetic neuropathy or other types of neuropathies are at increased risk of developing a higher grade following chemotherapy with cytostatic agents [33], there are no published reviews on the effect of chemotherapy administration in diabetic patients who do not have neuropathy or the evolution of neuropathy during cancer treatment in diabetic patients. The incidence of chemotherapy-induced peripheral neuropathy depends largely on the type of agent used, the duration of treatment (number of chemotherapy cycles received) and the dose used. In the case of diabetic patients it has been postulated that the loss of axonal integrity due to decreased regeneration makes diabetic patients prone to neuronal toxicity from drugs such as chemotherapy [34].

Among the pharmacological agents that have the greatest ability to induce peripheral neuropathy in oncological patients, taxanes and platinum derivatives have been the most studied [4]. For example, in treatment with the oxaliplatin derivative, a drug widely used in colorectal and other cancers, neurotoxicity usually presents mainly as peripheral sensory neuropathy, which can become persistent and therefore can be a dose-limiting toxicity of oxaliplatin or even treatment discontinuation. Persistent peripheral sensory neuropathy is cumulative with doses received, and in the case of oxaliplatin for example, severe neuropathy (Grade 3 and 4) occurs in 10–20% of patients receiving total doses of this drug from >750–850 mg/m^2^ [35]. Paclitaxel (taxanes family) induced peripheral neuropathy usually begins with paresthesia and numbness and is also cumulative and may result in patient functional impairment and limitation of use [36]. 

It is well known that diabetic patients with neuropathy have a lower quality of life, an increased risk of falls [37], and are at increased risk of ulcerations, which in turn can lead to lower extremity amputation [38]. Although in some cases symptoms may improve with appropriate glycemic control, a significant number of patients continue to experience neuropathic symptoms many years after the end of treatment [3].

In this review, we highlight the effects of chemotherapy in the onset or progression of neuropathy in diabetic patients, the type of chemotherapeutic drugs studied, and the forms of neuropathy (sensory, motor, and autonomic) most affected by chemotherapy in diabetic patients. Eventual treatment of diabetic patients to achieve lower or perhaps similar levels of neuropathic symptoms compared to patients without diabetes is also reviewed.

## 2. Methods

### 2.1. Literature Search

We searched the literature in the electronic bibliographic databases Medline and Scopus, covering all entries until 31 January 2021. As the first step in the scoping review, we established the standard terms related to the areas of knowledge that we wished to study, which would lead us to build the search equation. We used the MeSH (Medical Subject Headings) thesaurus to that end. After constructing the equation, we searched two databases: Medline through PubMed, and Scopus. The standard terms found in the thesaurus were: “peripheral neuropathy”, “antineoplastic agents”, which encompassed the term “chemotherapy”, and finally, “diabetes mellitus”. The search equation was therefore as follows:

- PubMed: (((antineoplastic agents) AND (diabetes mellitus)) AND (peripheral neuropathy)) NOT (review)
- Scopus: “antineoplastic agents” AND “diabetes mellitus” AND “peripheral neuropathy” AND NOT review AND NOT animals

The filters that were added to the search were the following:

- Studies carried out in humans.
- Articles of the type: review, meta-analysis and systematic review were excluded.
- Studies available in English or Spanish.

The searches were carried out in January 2021. A first search of PubMed using the pre-established equation and the indicated filters obtained 166 results. When the same equation was applied in Scopus, 47 documents were located, and although the filter of “studies carried out in human subjects” could not be applied in this database, this limit was included as part of the search equation. The search carried out in PubMed was then enriched by adding subheadings with the operator AND to the proposed Equation (3): “antineoplastic agents/toxicity”, “antineoplastic agents/adverse effects”, and “peripheral nervous system diseases/chemically induced”. This obtained five results. This second search could not be performed in Scopus, because this database does not permit subheadings to be added to the search strategy. To complete the search, a free query was proposed, which obtained 20 results in PubMed and 171 in Scopus, using the following search equations:

- PubMed: (((“diabetes mellitus”[All Fields]) AND (“chemotherapy”[All Fields])) AND (“neuropathy”[All Fields])) NOT (review)
- Scopus: “diabetes mellitus” AND chemotherapy AND “peripheral neuropathy” AND NOT review AND NOT animals

**Figure 1:**
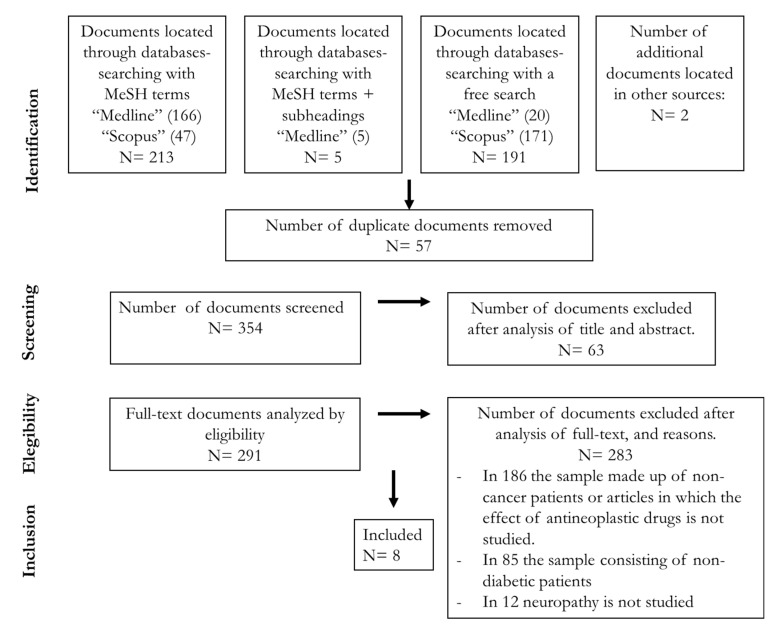
Systematic review workflow.

Considering the results obtained following the searches in the two databases, 411 articles were found, and eight articles were finally included in this scoping review (Figure 1).

### 2.2. Inclusion and Exclusion Criteria

The following inclusion criteria were used to review the scientific literature: (1) original articles based on both observational and experimental studies; (2) articles published in English or Spanish; (3) articles in which neuropathy was specifically evaluated with clinical anamnesis, physiological methods and/or specific questionnaires to assess the presence and severity of neuropathy; (4) articles in which chemotherapy-induced neuropathy was analyzed in diabetic patients (of any type) compared to patients without diabetes.

### 2.3. Analysis

The results of the database searches were first analyzed to eliminate duplicate references. Two team members independently reviewed the title and abstracts of the articles extracted from the literature search to determine which studies were to be included in the review. The electronic full text of studies on which the reviewers agreed was retrieved based on the inclusion/exclusion criteria mentioned above. Two reviewers independently extracted the following data for each selected article: number of participants, age and sex of participants, type of tumor, type of chemotherapy received, tools used for neuropathy assessment, effects on chemotherapy-induced neuropathy in diabetic versus non-diabetic patients, and the main outcomes of the studies. Any disagreement between the two reviewers regarding the articles and the data extracted from them was resolved by the third author.

## 3. Results

A total of 259 papers were retrieved from the studies identified by our search strategy. After eliminating duplicates, 26 met the inclusion criteria and were analyzed in detail. Finally, 8 articles [3,5,36,39,40,41,42,43] fulfilled the criteria (Figure 1). Four more articles were retrieved from the references of the selected articles although the data on neuropathy-induced by chemotherapy and diabetes were not the primary outcomes [4,44,45] or the study evaluated hyperglycemia (and not diabetes) [13]. Most studies were excluded because they did not compare the presence and/or severity of neuropathy between patients with or without diabetes or did not study the neuropathy in diabetic patients before and after chemotherapy administration. Two researchers independently summarized the results which emerged from this literature review under four headings: (1) characteristics of the studies; (2) prevalence and severity of neuropathy in diabetic patients compared to non-diabetic patients after chemotherapy; (3) severity of neuropathy and type of chemotherapeutic drugs in diabetic versus non-diabetic patients; (4) variables affecting the relationship between occurrence and severity of neuropathy in diabetic patients. 

### 3.1. Characteristics of the Studies Analyzed

The analysis of the studies covered the information about neuropathy recorded in 768 cancer patients with diabetes. Seven articles included data on control (non-diabetic patients) cancer patients, which amounted to 5247 cases [3,5,36,39,40,41,42]. The main features of the selected studies are shown in Table 2. Most of the studies evaluated the outcomes of the review in patients with colorectal cancer [3,5,39,41,42], in breast cancer patients [36], with ovarian cancer [43] or with no cancer [40]. As for the drugs evaluated for neuropathy-induced chemotherapy, the selected studies included treatment with paclitaxel [36], with a single-taxanes agent docetaxel, paclitaxel, or with a combination of platinum agents [40], oxaliplatinum containing different chemotherapy regimens [3,5], a FOLFOX regimen including the drugs leucovorin calcium (folinic acid), fluorouracil and oxaliplatin [5,39,41,42], cisplatin-based regimen and/or a taxol-based regimen [43].

### 3.2. Neuropathy Prevalence/Incidence after Chemotherapy in Diabetic Patients

Most of the studies found some effect of comorbid diabetes on neuropathy, ranging from clear and robust to subtle effects. For instance, in the free response questions evaluated in the study by Vissers et al. (2015), ≤3% of diabetic and non-diabetic patients responded to be “highly affected” in terms of sensory symptoms. The answer “a little” was more frequent: between 2 and 4% responded “a little” regarding problems with distinguishing the temperature of water, and between 27% and 33% reported hearing problems, in non-diabetic and diabetic patients respectively [3]. However, the probability of having neuropathic symptoms was higher among diabetic cancer patients than in non-diabetic patients in multivariate logistic regression models after adjustments for BMI, number of comorbidities, educational level, and cancer treatment (surgery, radiotherapy, and chemotherapy [3]). In patients with ovarian cancer, the pretreatment incidence of neuropathy was 39%. The overall incidence of neuropathy in the diabetic patient population treated with cisplatin was 67%, and three progressed to Grade 3 neuropathy [43].

In contrast, following administration of chemotherapy regimens with taxanes, diabetes patients showed a significantly higher incidence of peripheral neuropathy than non-diabetic patients [36,40]. For instance, diabetic patients developed peripheral neuropathy more frequently than non-diabetic patients during treatment with paclitaxel (74.4% vs. 58.4%), and its severity once developed was also greater (Grade 2–3: 51.2% versus 27.7%). Diabetic patients developed peripheral neuropathy more frequently than non-diabetic patients during treatment with paclitaxel (74.4% vs. 58.4%). The severity of peripheral neuropathy was also greater in diabetic patients (Grade 2–3: 51.2% versus 27.7%) but it was observed as being significantly higher in diabetics when all grades of neuropathy were considered [36]. Among several confounding factors such as age over 65 years, functional status, initial dose level, total dose, or between the number of chemotherapy cycles, only diabetes mellitus was a significant risk factor for paclitaxel-induced peripheral neuropathy. After a follow-up of 23.5 months, 81.8% of the diabetics developed peripheral neuropathy, while in the non-diabetics it occurred in 40.9%. Resolution of peripheral neuropathy was reported as delayed more frequently in patients with diabetes, and in a multivariate analysis, it was the only predictor of delayed recovery. In women with diabetes, peripheral neuropathy caused more delays in chemotherapy (20.9% vs. 7.1%) and dose reductions (32.6% vs. 11.9%) which can ultimately influence the efficacy of chemotherapy and quality of life of patients [36].

As a factor influencing the onset of chemotherapy-induced neuropathy, the duration of diabetes, has been investigated for taxanes chemotherapy. When considering the presence or absence of diabetes, the rate of taxanes-induced chemotherapy was similar among non-diabetics (48.8%) and diabetics with a duration of diabetes less than five years (52.8%), while in contrast, in diabetic patients with more than five years of evolution, the rates of neuropathy were significantly higher (75%) compared to non-diabetic patients [40]. However, the stronger effect was not observed in the group of patients treated only with taxanes (45.5% of the non-diabetics and 52.5% of the diabetics developed neuropathy) but in those patients treated with a combined regimen of taxanes and platinum e.g., 57.3% of non-diabetics and 81.8% of diabetics developed neuropathy with an OR 1919). The use of taxanes chemotherapy in diabetics with a disease duration of five years or more, therefore, increases the incidence and severity of peripheral neuropathy in patients without any known baseline neuropathy. The data showing the independent association of diabetes with paclitaxel-induced neuropathy incidence partially agree with previous studies: Nurgalieva et al. (2010) [4] did not find relevant differences in this outcome after stratification by diabetes (hazard ratio 2.92 vs. 3.33 for diabetes) in a large cohort of elderly patients treated with taxanes and/or platinum. Another retrospective study including 219 patients treated with adjuvant paclitaxel (mostly with the every-three-weeks schedule) found neither a longer duration nor a higher grade of paclitaxel-induced neuropathy in patients with diabetes, although their series only included 19 women with diabetes [45]. The same limitation (only five patients with diabetes) applies to the study by Kanbayashi et al. (2013) [44] in which no association was found between diabetes and paclitaxel-induced neuropathy incidence.

Among patients with diabetes, no differences in the incidence of weekly paclitaxel-related peripheral neuropathy were found for the type of treatment e.g., insulin vs. oral agents vs. diet) [36,43]. The development of clinically significant neuropathy (Grade 2 and greater) did not appear to be affected by age or sex [5].

### 3.3. Effects of Diabetes on Different Types of Neuropathic Alterations Induced by Chemotherapy

When looking at different neuropathy symptoms, including the studies that found no significant differences in neuropathy onset following chemotherapy between patients with or without comorbid diabetes [39], we observed significant effects for some of the neuropathy symptoms but not at all of them, although there were differences between the studies [3,39,42]. The study by Bano and Ikram (2019) [39] found no differences in the frequency and appearance of neuropathy symptoms such as hypoesthesia or taste alteration. However, the incidence of paresthesia was significantly higher in diabetic patients with colorectal cancer treated with the FOLFOX regimen frequently reported in diabetic cancer patients but the risk of frequent, distal and transient paresthesia was studied shortly after oxaliplatin infusion (FOLFOX regimen) [39]. Dizziness is more prevalent in diabetic patients, and particularly in those treated with the FOLFOX regimen. Results obtained in a study based on a large database analysis of clinical records showed that comorbid diabetes affects several neuropathy symptoms and, in multivariate logistic regression analysis after adjusting the outcomes for confounding factors such as body mass index, the number of comorbidities and the type of cancer treatment received, it was shown that patients with diabetes were more likely to report neuropathy symptoms of any intensity and of various types such as tingling in the fingers or hands, tingling in the toes or feet, numbness in the toes or feet and erection problems among men [3]. In contrast, in the study by Abdel-Rahman (2018), conducted in patients with metastatic colorectal cancer, no significant differences were obtained between patients with and without diabetes in terms of neuropathy induced by oxaliplatin administration. This lack of differences included various neuropathic symptoms such as increased sensitivity to cold, laryngeal dysesthesia, and neuropathic pain. In addition, there were no differences between the two groups in terms of the incidence of long-term oxaliplatin-induced paresthesia, the highest level of paresthesia, or rates of recovery from paresthesia. However, no adjustment for confounding factors was made in the latter study [42].

### 3.4. Neuropathy Onset and Influence on Chemotherapeutic Drugs and Dosage in Diabetic Patients with Cancer

There was a significant relationship between diabetes mellitus and the cumulative dose at the onset of neuropathy in colorectal cancer patients [5], but no differences were reported [41]. The mean cumulative dose of oxaliplatin in which patients developed neuropathy was significantly lower in the diabetic group (338 mg/m^2^) than in the non-diabetic group (610 mg/m^2^) [5]. Most patients were unlikely to develop neuropathy until the fourth cycle of chemotherapy. However, from the fifth cycle of chemotherapy, patients with diabetes were more likely to develop it than patients without diabetes. The probability of peripheral neuropathy by cumulative dose of oxaliplatin was similar for colorectal cancer patients with and without diabetes [41]. In the MOSAIC study, peripheral neuropathy Grade 1 was reported in 93% and 92% of patients with and without diabetes and Grade 3 was reported in 12% and 13% respectively, with no statistically significant effect depending on the oxaliplatin dose in patients with or without diabetes [41]. No differences were reported between diabetic and non-diabetic patients regarding the treatment of colorectal cancer [3], and as such, the differences regarding neuropathic symptoms cannot be due to differences in chemotherapy treatment. In multivariate logistic regression models adjusted for sex, age at the time of cancer diagnosis, cancer stage, and excluding first-stage cancer patients, there was no association between diabetes mellitus and chemotherapy, or radiotherapy, or oxaliplatin [3,41].

### 3.5. Diabetes Control and Treatment during Chemotherapy Treatment

Only one study reported data on glycemic control in cancer patients with diabetes treated with chemotherapy [43]. The effect reported concerns the hyperglycemic effect of dexamethasone to prevent paclitaxel allergic reaction in patients with ovarian cancer and comorbid diabetes. Glucose levels were monitorized 30 min prior to the infusion of paclitaxel and after administration of 20 mg of dexamethasone administered orally approximately 12 and 6 hr prior the administration of paclitaxel. Two patients (out 18, 11.1%) required insulin after starting paclitaxel and three others (16.7%) oral hypoglycemic therapy after being managed with diet alone [43]. In a large study performed in breast cancer patients, it has been showed that glycemic instability was associated with an increased risk of neuropathy after adjuvant taxanes administration, but unfortunately, information regarding a pre-existing history of diabetes was not available in this study [13]. 

One study reported that among breast cancer patients with diabetes, no differences in the incidence of weekly paclitaxel-related peripheral neuropathy were found for the type of treatment (insulin vs. oral agents vs. diet) [36]. In patients with advanced colorectal cancer patients treated with different FOLFOX regimens those with diabetes were maintained on required doses of insulin during the course of treatment but the extent of insulin adjustment during treatment was not specified [39].

## 4. Discussion

Our analysis reveals that most neuropathic symptoms or the onset of neuropathy occurs at an early stage [42] or at lower doses [5] in diabetic patients than in non-diabetic patients treated by chemotherapy. Moreover, among diabetic patients receiving chemotherapy, neuropathy rates were higher in patients with a longer duration of diabetes [40]. However, this deleterious effect was not associated with differences in cancer treatment including chemotherapy, which suggests that diabetes mellitus rather than chemotherapy could be responsible for these symptoms. In addition, the effects seem to last longer, since two years after treatment, peripheral neuropathy persisted in a higher proportion of diabetic patients than in non-diabetic patients (68.7% vs. 29.2%). Furthermore, it was a functionally significant peripheral neuropathy in 18.2% of the cases [36]. Since not all chemotherapeutic drugs have the same ability to cause peripheral neuropathy, the drugs investigated are chemotherapy drugs with a high potential to induce neuropathy, such as platinum compounds and taxanes, which are currently the mainstay of treatment for various common tumors. Clinical studies report incidences of neuropathy after chemotherapy of up to 60% with cisplatin, paclitaxel, docetaxel and oxaliplatin, among other drugs [50,51,52]. In particular, oxaliplatin, which is widely used in the treatment of colon cancer, causes acute and transient neurotoxicity in almost all patients treated, and subsides after about 48 h [19,53], and becomes chronic in a few cases [50]. Some of these studies mention that the risk of paresthesia is increased in diabetic patients during the first minutes of infusion [39] and development takes place in a shorter time or with lower cumulative doses compared with non-diabetic patients [5,42]. Future studies should evaluate the influence of the severity and duration of diabetes. As for acute chemotherapy-induced neurological symptoms, Abdel-Rahman (2018) [42], found no differences between diabetic and non-diabetic patients, although diabetic patients developed oxaliplatin-induced paresthesia in a shorter time. Vissers et al. (2015) [3] concluded that diabetic patients suffered a higher burden of neuropathic symptoms than non-diabetic patients, regardless of the antineoplastic treatment. 

The differences between the results obtained in the studies analyzed may have been influenced by the fact that the first studies [5,36,39,40,41,42,43] used diagnostic tools such as scales to assess peripheral neuropathy, which usually focus on more severe degrees, while the latter study used a questionnaire to help detect milder symptoms [3]. Chemotherapy regimens based on taxanes frequently used in the treatment of different types of cancer such as lung, breast, ovarian and pancreatic cancer, among others, also present a high incidence of neuropathy. Within these regimens, paclitaxel affects 57–83% of patients, if any degree of neuropathy is considered, and 2–33%, in the case of severe neuropathies [50]. It usually causes axonal neuropathy with sensory loss of glove-sock distribution, pain, dysesthesia, and paresthesia, including nerve hyperexcitability. An earlier study by Gogas et al. (1996) [43], concluded that neurotoxicity came from the drug itself (67% incidence with platinum agents and 50% with paclitaxel) and that the presence of diabetes did not increase the incidence [43]. In this study, a little more than a third of the sample had a pre-existing neuropathy, different stages of diabetes, and the patients themselves had heterogeneous characteristics that were not considered. 

Subsequent studies that have also worked directly with the diabetic population have observed that diabetes acts as a risk factor in terms of the incidence and severity of peripheral neuropathy in those patients treated with chemotherapy [36,40]. Kus et al. (2016) [40] jointly evaluated the influence of taxanes and their combination with platinum derivatives and found that the incidence was higher in diabetic patients who received the combination of both agents. They also grouped patients by degree of hyperglycemia, but this did not show any difference in the development of peripheral neuropathy [40].

At present it is known that chemotherapy-induced neuropathy interferes with the quality of life and it is often accompanied by depressive symptoms and apathy and abandonment of leisure activities and physical activity [49,54,55]. It should be taken into account that the symptoms of neuropathy can last between two to eleven years after its diagnosis in more than half of the patients, where tingling and numbness affect 70% of the patients with chemotherapy-induced peripheral neuropathy [55] and those patients displaying higher neuropathy symptoms also showed a reduced quality of life after chemotherapy discontinuation as recently reported for taxanes in patients with breast cancer [56] and for oxaliplatin in colorectal cancer patients [57] or carboplatin/taxanes regimen in patients with gynecologic cancer survivors [58]. Regarding diabetic patients and chemotherapy-induced neuropathy in terms of health-related quality of life, the study by Visser et al. by using the European Organization for Research and Treatment of Cancer quality of life questionnaire—chemotherapy-induced peripheral neuropathy (EORTC QLQ-CIPN20) evaluated the differences in neuropathic symptoms between colorectal cancer patients with and without diabetes. Those with diabetes reported a decreased quality of life associated with higher impairment in sensitive symptoms such as tingling in fingers, hands, toes and feet, numbness, aching or burning pain in toes or feet, and trouble while standing or walking. In contrast, no differences were reported for neuropathic symptoms regarding motor symptoms or autonomic dysfunction except for getting or maintaining an erection in men [3]. 

In patients with diabetes, corticosteroids are generally avoided, as they can result in deterioration of glycaemic control and its consequences including neuropathy [59,60]. However, corticosteroids are widely used as anti-emetics preventive treatment in many chemotherapy regimens [61,62,63] and even they are used few times for this purpose that can worsen neuropathy symptoms in diabetic patients or they could contribute to the occurrence of neuropathy symptoms in diabetic patients. More importantly, many chemotherapy regimens include corticosteroids drug in the protocol administration of chemotherapy. For instance, taxanes widely used in the management of common cancers such as breast, lung, and ovarian, in both early and advanced disease settings and for many cancers in advanced stages used the corticosteroid drug dexamethasone as premedication to reduce taxanes-induced severe hypersensitivity reactions [64]. The corticosteroid drug, prednisone, is co-administered with abiraterone a drug inhibiting an enzyme necessary for androgen synthesis for the treatment of metastatic castration-resistant prostate cancer [65] and corticosteroids are included in many chemotherapy regimens for treating in many hematological malignancies [66]. Corticosteroid treatment can cause myopathy [67] and it can worsen neuropathy [68]. Hyperglycemia, resulting from use of dexamethasone in women with advanced ovarian cancers that received the corticosteroid pre-treatment to prevent taxanes adverse effects [43], is a frequent side effect. Glucose levels over 19.2 mmol/liter (350 mg/dl) were reported in the majority of patients and some of them required treatment change for better diabetes control [43]. Future studies should address if these associations can exacerbate neuropathy or induce neuropathy in diabetic patients with cancer. The role of corticosteroid treatment deserves future clinical studies on neuropathy occurrence after chemotherapy in diabetic patients. For many patients, when signs of severe chemotherapy-induced peripheral neuropathy appear, dose reduction or treatment cessation is usually implemented, thereby limiting the efficacy of cancer treatment [51,55,69]. The analysis of delay/discontinuing chemotherapy in diabetes patients for the occurrence of severe neuropathy was reported in two studies [36,43]. In women with breast cancer and comorbid diabetes, paclitaxel-induced peripheral neuropathy caused more chemotherapy delays (20.9% vs. 7.1%) and dose reductions (32.6% vs. 11.9%) compared to patients without diabetes [36]. No discontinuation of therapy due to neuropathy was required in patients with ovarian cancer or breast cancer treated with paclitaxel and/or cisplatin [36,43] and no patient had evidence of drug-induced autonomic neuropathy, such as orthostatic hypotension, generalized weakness, or paralytic ileus. Besides diabetes, other factors often associated with diabetes, might play role on the occurrence of neuropathy or its worsening after chemotherapy administration. Apart from glycemic control, the incidence of neuropathy is diabetic patients associated with potentially modifiable cardiovascular risk factors, including a raised triglyceride level, body-mass index, smoking, and hypertension [70,71]. In the case of cancer chemotherapy-induced neuropathy, obesity has been associated with an increased risk of neuropathy in breast cancer patients who received taxane-based chemotherapy [13,72]. Patients with complications from diabetes had more than twice the odds of having neuropathy compared with patients with no diabetes [12]. In contrast, hypertension and hypercholesterolemia do not seem to play a major role in chemotherapy-induced neuropathy [12].

The lack of standardized instruments to grade peripheral neuropathy valid for all chemotherapy drugs may pose a problem when investigating the possible influence of diabetes on this issue, as they vary widely, and there are no specific guidelines for their use [73]. Physical assessment and monitoring of subjective symptoms of peripheral neuropathy are important in the early detection of chemotherapy-related neurotoxicity, and both objective and subjective tools are warranted for comparisons. Evaluation of a diabetic patient with cancer should begin with a detailed analysis of neuropathy symptoms present prior to the initiation of treatment because cancer per se can induce neuropathy signs [73,74,75]. Other limitations of the analysed studies concern their retrospective nature about clinical data related to diabetes treatment and glycemic control. The details regarding the duration and type of diabetes, medications, or information about diet before and after chemotherapy administration were lacking in most studies. One study reported that among breast cancer patients with diabetes, no differences in the incidence of weekly paclitaxel-related peripheral neuropathy were found for the type of treatment (insulin vs. oral agents vs. diet) [36]. Gogas et al. (1995) [43] conclude the paclitaxel/cisplatin combination regimen or paclitaxel alone could be safely administered in diabetic patients at standard doses, with concurrent glucose monitoring in order to adjust pharmacological treatment of diabetes (or hyperglycemia as surrogate of diabetes control). Since the principal pathophysiological mechanisms leading to neuropathy development in diabetic patients are oxidative stress, inflammation and microangiopathy (*vasa nervorum*) [76,77,78,79,80] future studies are warranted to identify the mechanisms by which chemotherapy drugs synergize with diabetes-induced nerve damage in order to tailor treatments to minimize their occurrence or reduce their impact in diabetic patients with cancer.

Future studies investigating the risk of neuropathy in cancer patients with comorbid diabetes need to be analyzed considering the duration of diabetes, cancer-induced neuropathic effects per se, prior cancer management strategies such as radiotherapy and surgery, and the type and dose of chemotherapy used.

## Figures and Tables

**Table 1:** Incidence of neuropathy induced by main classes of cancer chemotherapy drugs.

Class of Chemotherapy Drugs	Drug Name	Neuropathy Incidence (%) (Classified by Neurotoxicity Degrees)	Reported Neurotoxic Doses
Anthracyclines	Doxorubicin (Adriamycin)	75% (cognitive impairment “chemobrain”) [14]	-
Taxanes	Paclitaxel	All grades: 60% [15] Grade 3–4 motor: 11% Grade 3–4 sensory: 33% [16]	1000 mg/m^2^ cumulative dose [17]
	Docetaxel	All grades: 15% [15,18] Grade 3–4: 2% [18]	400 mg/m^2^ cumulative dose [17]
Platinum-based agents	Cisplatin	Grade 1:14–33% Grade 2: 0–33% Grade 3: 2–19% Grade 4: 0–4% [19]	250–450 mg/m^2^, and all patients develop neuropathy [19,20], at cumulative dose of 500–600 mg/m^2^ [21]
	Oxaliplatin	Grade 1: 21–94% Grade 2: 5–42% Grade 3–4: 3–19% [19,22]	>550 mg/m^2^ Severe neurotoxicity at cumulative dose of 750–850 mg/m^2^ [23] Chronic neuropathy with a cumulative dose between 850 mg/m^2^ and 1800 mg/m^2^ [19]
	Carboplatin	All grades: 4–6% [24]	>400 mg/m^2^ Neurotoxicity only with high doses or in combination with other drugs [20]
Vinca alkaloids	Vincristine	Grade 1–2: 60% [15]	30–50 mg [15,20]
	Vinorelbine	All grades: 44% [25] Grade 3–4: ≈ 2% [26]	125 mg/m^2^ [25]
Antimetabolites	5-fluorouracil (5-FU)	All grades: 0.6–7% [27,28] or 12.9% [29] Grade 3–4: 0% [29]	Uncertain [30] High doses and use of 5-FU in combined treatment, increase the risk of neuropathy [31]
	Gemcitabine	All grades: 6% [32]	-
	Methotrexate	All grades: 3–10% [31]	-

**Table 2:** Peripheral neuropathy induced by chemotherapy: differences between selected studies.

Reference	Population Characteristics			Number of Patients		Type of Cancer and Chemotherapy Treatment	Measurement of Peripheral Neuropathy	Main Outcomes
				DM	No DM			
Bano and Ikram 2019 [39]	N	38		6	32	Colorectal cancer with metastasis Treatment: FOLFOX	Oxaliplatin Specific Neurotoxicity Scale (OSNS) [46] National Cancer Institute Common Toxicity Criteria 2.0 (NCI-CTC) [47]	It cannot be confirmed that PN induced by FOLFOX chemotherapy has a higher incidence in diabetics than in non-diabetics. However, dizziness is more common in diabetic cancer patients than in those without diabetes mellitus. Distal and transitory paresthesia after administration of oxaliplatin in patients with colorectal cancer are also more prevalent in the group of diabetic subjects.
	Country	Pakistan						
	Gender (%)	Unknown						
	Age (range)	Total: 20–80						
De la Morena Barrio et al. 2015 [36]	N	129		43	86	Breast cancer Treatment: paclitaxel	National Cancer Institute Common Toxicity Criteria (NCI-CTC) [47]	An increased incidence of PIPN was observed in women with breast cancer with diabetes mellitus. A substantial delay in recovery was also observed, as well as a greater frequency and severity of peripheral neurotoxicity in diabetic patients than in non-diabetic patients treated with this chemotherapy drug.
	Country	Spain						
	Gender (%)	♀	100%					
		♂	0%					
	Age (mean)		Total: 66					
			DM: 66					
			No DM: 65.5					
Kus et al. 2016 [40]	N	374		81	293	Any cancer Treatment: taxanes (docetaxel or paclitaxel) for one group and in combination with platinum for another group.	Neuropathic Pain Symptom Inventory (NPSI) [48] Clinical criteria	The incidence of PSN among cancer patients treated with taxanes was higher in patients with diabetes mellitus. The neuropathy rate was similar between non-diabetic and diabetics with less than 5 years of evolution, while those with diabetes of more than 5 years had higher neuropathy rates. The presence of diabetes with more than 5 years of evolution influences the incidence and severity of PSN in cancer patients treated with taxanes.
	Country	Turkey						
	Gender (%)	♀	97%					
		♂	3%					
	Age (*n*)	Total: <65 years: 337 ≥65 years: 37						
		DM <65 years: 72 ≥65 years: 9						
		No DM <65 years: 265 ≥65 years: 28						

Ramanathan et al. 2009 [41]	N	3430		309	3121	Colorectal cancer Treatment: FOLFOX, fluorouracil and leucovorin, or oxaliplatin	National Cancer Institute Common Toxicity Criteria (EFC2962 and MOSAIC) [47] and Oxaliplatin Specific Neurotoxicity Scale (EFC4584) [46].	The presence of diabetes was not associated with an increased risk of developing PSN in any of the patients in the total sample. Diabetes did not affect the incidence, severity, and time to onset of peripheral neuropathy in patients with colon cancer treated with oxaliplatin.
	Country	Trials with different samples: 1. International (MOSAIC) 2. North American (EFC4584) 3. European (EFC2962)						
	Gender (%)	♀	44.4%					
		♂	55.6%					
	Age median	DM: 63						
		No DM: 60						
Uwah et al. 2012 [5]	N	62		14	48	Colorectal Cancer Treatment: oxaliplatin	National Cancer Institute Common Toxicity Criteria (NCI-CTC) [47]	No influence of diabetes mellitus on the severity of oxaliplatin-induced peripheral neuropathy was found. However, patients with diabetes developed neuropathy with a lower cumulative dose of oxaliplatin than non-diabetic patients.
	Country	United States						
	Gender (%)	♀	50.2%					
		♂	49.8%					
	Age (mean)	Total: 60.2						
Abdel-Rahman 2018 [42]	N	756		64	692	Colorectal cancer with metastasis Treatment: FOLFOX	Neurological symptoms	Diabetes did not affect the overall survival of patients with colorectal cancer treated with FOLFOX. However, diabetic patients appear to be more predisposed to developing oxaliplatin-induced peripheral neuropathy in a shorter time than non-diabetic patients, although diabetes did not influence the incidence or rate of recovery from peripheral nerve disease.
	Country	Unknown						
	Gender (%)	♀	40.2%					
		♂	59.8%					
	Age (mean)	Total: 60.5						
		DM: 64.4						
		No DM: 60.1						

Gogas et al. 1996 [43]	N	33		33	0	Celomic epithelial ovarian cancer Treatment: paclitaxel and/or cisplatin	National Cancer Institute Common Toxicity Criteria (NCI-CTC) [47]	The treatment of paclitaxel and cisplatin in combination, or paclitaxel alone, can be administered safely in diabetic patients using a standard dose and with simultaneous monitoring of blood glucose and creatinine. It must be accompanied by a previous study of the history of the patient’s neurological symptoms and a physical examination.
	Country	United States						
	Gender (%)	♀	100%					
		♂	0%					
	Age (mean)	Total: 61						
Vissers et al. 2015 [3]	N	1193		218	975	Colorectal cancer	European Organization for Research and Treatment of Cancer quality of life questionnaire-chemotherapy-induced peripheral neuropathy 20 (EORTC QLQ-CIPN20) [49]	Diabetic patients with colorectal cancer showed neuropathic symptoms more frequently than non-diabetic patients regardless of chemotherapy treatment, which suggests that diabetes mellitus rather than chemotherapy is the main factor responsible for neuropathic symptoms in colorectal cancer patients.
	Country	Netherlands						
	Gender (%)	♀	33%					
		♂	67%					
	Age (mean)	Total: 71						
		DM: 71.3						
		No DM: 70.8						


PN: peripheral neuropathy; PSN: peripheral sensory neuropathy; PIPN: paclitaxel-induced peripheral neuropathy; DM: Diabetes Mellitus.
